# *N*-[7-Eth­oxy-1-(prop-2-en-1-yl)-1*H*-indazol-4-yl]-4-methyl­benzene­sulfonamide

**DOI:** 10.1107/S1600536811019465

**Published:** 2011-06-04

**Authors:** Najat Abbassi, El Mostapha Rakib, Hafid Zouihri

**Affiliations:** a Laboratoire de Chimie Organique et Analytique, Université Sultan Moulay Slimane, Faculté des Sciences et Techniques, Béni-Mellal, BP 523, Morocco; b Laboratoires de Diffraction des Rayons X, Centre Nationale pour la Recherche Scientifique et Technique, Rabat, Morocco

## Abstract

In the title compound, C_19_H_21_N_3_O_3_S, the C—SO_2_—NH—C torsion angle is 103.72 (11)°. The almost planar indazole ring [r.m.s. deviation = 0.0202 (14) Å] is twisted away from the methyl­benzene ring by 76.87 (7)°. The vinyl group is disordered over two orientations with site occupancies of 0.622 (10) and 0.378 (10). The S atom has a distorted tetra­hedral geometry [maximum deviation: O—S—O = 119.18 (11)°]. An intra­molecular C—H⋯O hydrogen bond occurs. In the crystal, two mol­ecules are linked about a center of inversion by pairs of  N—H⋯O hydrogen bonds, generating a dimer. C—H⋯π inter­actions are also observed.

## Related literature

For a related structure, see: Abbassi *et al.* (2011*b* ▶). For the biological activity of sulfonamides, see: Soledade *et al.* (2006 ▶); Lee & Lee (2002 ▶). For the synthesis of 7-eth­oxy-*N*-alkyl­indazole derivatives, see: Abbassi *et al.* (2011*a* ▶).
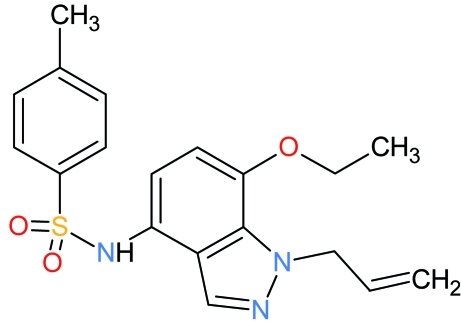


## Experimental

### 

#### Crystal data

C_19_H_21_N_3_O_3_S
*M* *_r_* = 371.45
Triclinic, 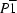

*a* = 8.2208 (3) Å
*b* = 10.4985 (4) Å
*c* = 11.9655 (5) Å
α = 108.814 (2)°
β = 92.346 (2)°
γ = 107.500 (2)°
*V* = 921.33 (6) Å^3^
*Z* = 2
Mo *K*α radiation
μ = 0.20 mm^−1^
*T* = 296 K
0.32 × 0.17 × 0.12 mm

#### Data collection

Bruker APEXII CCD detector diffractometer
23139 measured reflections
3629 independent reflections
3281 reflections with *I* > 2σ(*I*)
*R*_int_ = 0.023

#### Refinement

*R*[*F* ^2^ > 2σ(*F* ^2^)] = 0.042
*wR*(*F* ^2^) = 0.118
*S* = 1.08
3629 reflections
259 parameters
6 restraints
H atoms treated by a mixture of independent and constrained refinement
Δρ_max_ = 0.39 e Å^−3^
Δρ_min_ = −0.32 e Å^−3^

### 

Data collection: *APEX2* (Bruker, 2005 ▶); cell refinement: *SAINT* (Bruker, 2005 ▶); data reduction: *SAINT*; program(s) used to solve structure: *SHELXS97* (Sheldrick, 2008 ▶); program(s) used to refine structure: *SHELXL97* (Sheldrick, 2008 ▶); molecular graphics: *PLATON* (Spek, 2009 ▶); software used to prepare material for publication: *publCIF* (Westrip, 2010 ▶).

## Supplementary Material

Crystal structure: contains datablock(s) I, global. DOI: 10.1107/S1600536811019465/ng5164sup1.cif

Structure factors: contains datablock(s) I. DOI: 10.1107/S1600536811019465/ng5164Isup2.hkl

Supplementary material file. DOI: 10.1107/S1600536811019465/ng5164Isup3.cml

Additional supplementary materials:  crystallographic information; 3D view; checkCIF report

## Figures and Tables

**Table 1:** Hydrogen-bond geometry (Å, °) *Cg*1 is the centroid of the C7–C12 ring.

*D*—H⋯*A*	*D*—H	H⋯*A*	*D*⋯*A*	*D*—H⋯*A*
N3—H3⋯O3^i^	0.86 (2)	2.15 (2)	3.002 (2)	171 (2)
C14—H14*B*⋯O1	0.97	2.35	2.974 (2)	121
C19—H19*C*⋯*Cg*1^ii^	0.96	2.87	3.622 (2)	136

Symmetry codes: (i) 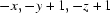; (ii) 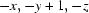.

**Fig. 1.**
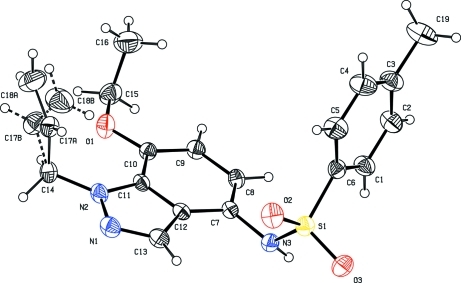
Molecular view of the title compound showing the atom-labeling scheme. Displacement ellipsoids are drawn at the 30% probability level. H atoms are represented as small spheres of arbitrary radii.

**Fig. 2.**
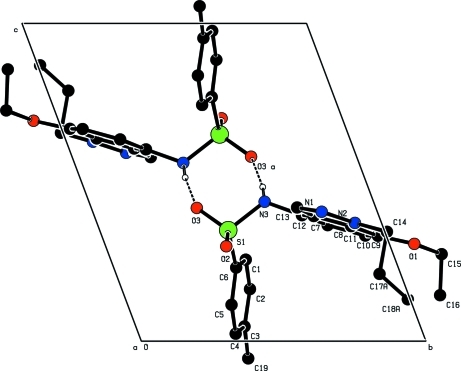
Partial packing view showing the chain formed by N—H···O. H atoms not involved in hydrogen bonds have been omitted for clarity.

## supplementary crystallographic information

### Comment

Various sulfonamides are widely used as anti-hypertensive [Soledade *et al.*, 2006; Lee & Lee, 2002]. In a former paper, we reported the crystal structure of *N*-(7-ethoxy-1*H*-indazol-4-yl)-4-methylbenzenesulfonamide [Abbassi *et al.*, 2011*b*]. In this communication, the crystal structure of *N*-[7-ethoxy-1-(prop-2-en-1-yl)-1*H*-indazol-4-yl]-4-methylbenzenesulfonamide is reported.

The title heterocyclic compound, C_19_H_21_N_3_O_3_S, is a new synthetic molecule whish is bent at the S atom with an C—SO~2~—NH—C torsion angle of 103.72 (11)°. The indazol planar ring [r.m.s. deviation: 0.0202 (14) Å] is twisted away from the methylbenzene ring by 76.87 (7)°. The vinyl group is disordered over two positions with site occupancies of 0.622 (10) and 0.378 (10). The S atom has a distorted tetrahedral geometry [maximum deviation: O—S—O = 119.17 (10)°].

In the crystal structure, the molecules are linked by N—H···O hydrogen bonds together with weak C—H···O interactions. There also exist C—H···*Cg* contacts between the methyl groups of the methylbenzene and the indazol rings. The crystal structure is further stabilized by intermolecular π–π stacking interactions [centroid–centroid distances = 3.6673 (9)–3.8109 (10) A °].

### Experimental

A mixture of 1-allyl-4-nitro-1*H*-indazole [Abbassi *et al.*, 2011*a*] (1.22 mmol) and anhydrous SnCl_2_ (1.1 g, 6.1 mmol) in 25 mL of absolute ethanol was heated at 60 °C for 2 h. After reduction, the starting material disappeared, and the solution was allowed to cool down. The pH was made slightly basic (pH 7–8) by addition of 5% aqueous potassium bicarbonate before extraction with ethyl acetate. The organic phase was washed with brine and dried over magnesium sulfate. The solvent was removed to afford the amine, which was immediately dissolved in pyridine (5 ml) and then reacted with 4-methylbenzenesulfonyl chloride (0.26 g, 1.25 mmol) at room temperature for 24 h. After the reaction mixture was concentrated *in vacuo*, the resulting residue was purified by flash chromatography (eluted with Ethyl acetate: Hexane 1:9).

### Refinement

The H atoms bound to C were positioned geometrically and constrained to ride on their parent atoms [C—H distances are 0.93Å for CH groups with *U*_iso_(H) = 1.2 *U*_eq_(C), and 0.97 Å for CH_3_ groups, and the N3—H3 atoms were refined with restraints (d_N–H_ = 0.86 (2) Å) and then were treated as riding in the last cycles of refinement. The vinyl group is disordered over two positions with site occupancies of 0.622 (10) and 0.378 (10), the corresponding C—C and C==C distances in the major and minor conformers were refined with distance restraints of: 1.54 (2) Å and 1.35 (2) Å, respectively.

### Crystal data

**Table:**

C_19_H_21_N_3_O_3_S	*Z* = 2
*M**_r_* = 371.45	*F*(000) = 392
Triclinic, *P*1	*D*_x_ = 1.339 Mg m^−^^3^
Hall symbol: -P 1	Mo *K*α radiation, λ = 0.71073 Å
*a* = 8.2208 (3) Å	Cell parameters from 341 reflections
*b* = 10.4985 (4) Å	θ = 2.5–27.9°
*c* = 11.9655 (5) Å	µ = 0.20 mm^−^^1^
α = 108.814 (2)°	*T* = 296 K
β = 92.346 (2)°	Prism, colourless
γ = 107.500 (2)°	0.32 × 0.17 × 0.12 mm
*V* = 921.33 (6) Å^3^	

### Data collection

**Table:**

Bruker APEXII CCD detector diffractometer	3281 reflections with *I* > 2σ(*I*)
Radiation source: fine-focus sealed tube	*R*_int_ = 0.023
graphite	θ_max_ = 26.0°, θ_min_ = 2.2°
ω and φ scans	*h* = −10→9
23139 measured reflections	*k* = −12→12
3629 independent reflections	*l* = −14→14

### Refinement

**Table:**

Refinement on *F*^2^	Primary atom site location: structure-invariant direct methods
Least-squares matrix: full	Secondary atom site location: difference Fourier map
*R*[*F*^2^ > 2σ(*F*^2^)] = 0.042	Hydrogen site location: inferred from neighbouring sites
*wR*(*F*^2^) = 0.118	H atoms treated by a mixture of independent and constrained refinement
*S* = 1.08	*w* = 1/[σ^2^(*F*_o_^2^) + (0.053*P*)^2^ + 0.4364*P*] where *P* = (*F*_o_^2^ + 2*F*_c_^2^)/3
3629 reflections	(Δ/σ)_max_ < 0.001
259 parameters	Δρ_max_ = 0.39 e Å^−^^3^
6 restraints	Δρ_min_ = −0.32 e Å^−^^3^

### Special details

**Table:**

Geometry. All e.s.d.'s (except the e.s.d. in the dihedral angle between two l.s. planes) are estimated using the full covariance matrix. The cell e.s.d.'s are taken into account individually in the estimation of e.s.d.'s in distances, angles and torsion angles; correlations between e.s.d.'s in cell parameters are only used when they are defined by crystal symmetry. An approximate (isotropic) treatment of cell e.s.d.'s is used for estimating e.s.d.'s involving l.s. planes.
Refinement. Refinement of *F*^2^ against ALL reflections. The weighted *R*-factor *wR* and goodness of fit *S* are based on *F*^2^, conventional *R*-factors *R* are based on *F*, with *F* set to zero for negative *F*^2^. The threshold expression of *F*^2^ > σ(*F*^2^) is used only for calculating *R*-factors(gt) *etc*. and is not relevant to the choice of reflections for refinement. *R*-factors based on *F*^2^ are statistically about twice as large as those based on *F*, and *R*- factors based on ALL data will be even larger.

### Fractional atomic coordinates and isotropic or equivalent isotropic displacement parameters (Å^2^)

**Table:**

	*x*	*y*	*z*	*U*_iso_*/*U*_eq_	Occ. (<1)
C1	−0.1369 (3)	0.4744 (3)	0.2566 (2)	0.0518 (6)	
C10	0.4698 (3)	0.9705 (2)	0.33160 (18)	0.0362 (4)	
C11	0.5617 (2)	0.8894 (2)	0.36197 (17)	0.0345 (4)	
C12	0.4804 (3)	0.7709 (2)	0.39361 (17)	0.0346 (4)	
C13	0.6165 (3)	0.7239 (3)	0.4207 (2)	0.0444 (5)	
C14	0.8737 (3)	1.0072 (3)	0.3443 (2)	0.0574 (7)	
C15	0.4773 (4)	1.1700 (3)	0.2738 (3)	0.0595 (7)	
C16	0.4069 (5)	1.1104 (4)	0.1447 (3)	0.0873 (10)	
C17A	0.8898 (7)	0.9302 (9)	0.2134 (5)	0.0553 (16)	0.622 (10)
C17B	0.9147 (12)	1.0054 (12)	0.2270 (8)	0.057 (3)	0.378 (10)
C18A	0.8938 (10)	0.9902 (9)	0.1303 (6)	0.101 (3)	0.622 (10)
C18B	0.8493 (13)	0.8767 (12)	0.1399 (10)	0.079 (4)	0.378 (10)
C19	−0.3856 (5)	0.3527 (4)	−0.0543 (3)	0.0891 (11)	
C2	−0.2614 (4)	0.4503 (3)	0.1640 (2)	0.0587 (7)	
C3	−0.2476 (4)	0.3819 (3)	0.0462 (2)	0.0596 (7)	
C4	−0.1046 (4)	0.3413 (3)	0.0237 (2)	0.0697 (8)	
C5	0.0215 (4)	0.3650 (3)	0.1146 (2)	0.0595 (7)	
C6	0.0038 (3)	0.4302 (2)	0.2315 (2)	0.0422 (5)	
C7	0.3012 (3)	0.7306 (2)	0.39635 (17)	0.0348 (4)	
C8	0.2116 (3)	0.8081 (2)	0.36507 (19)	0.0395 (5)	
C9	0.2950 (3)	0.9261 (2)	0.3323 (2)	0.0408 (5)	
H1	−0.1478	0.5201	0.3352	0.062*	
H13	0.6020	0.6459	0.4448	0.053*	
H14A	0.9800	1.0318	0.3969	0.069*	
H14B	0.8479	1.0937	0.3523	0.069*	
H15A	0.5564	1.2664	0.2933	0.071*	
H15B	0.3835	1.1747	0.3199	0.071*	
H16A	0.4981	1.0999	0.0987	0.131*	
H16B	0.3571	1.1738	0.1251	0.131*	
H16C	0.3198	1.0189	0.1266	0.131*	
H17A	0.8970	0.8388	0.1918	0.066*	0.622 (10)
H17B	0.9807	1.0864	0.2132	0.069*	0.378 (10)
H18A	0.8868	1.0814	0.1503	0.121*	0.622 (10)
H18B	0.9036	0.9407	0.0523	0.121*	0.622 (10)
H18C	0.7840	0.7984	0.1577	0.095*	0.378 (10)
H18D	0.8694	0.8659	0.0620	0.095*	0.378 (10)
H19A	−0.3360	0.3474	−0.1263	0.134*	
H19B	−0.4341	0.4283	−0.0346	0.134*	
H19C	−0.4746	0.2638	−0.0662	0.134*	
H2	−0.3559	0.4805	0.1810	0.070*	
H3	0.146 (3)	0.630 (3)	0.484 (2)	0.049*	
H4	−0.0927	0.2968	−0.0549	0.084*	
H5	0.1176	0.3372	0.0973	0.071*	
H8	0.0931	0.7822	0.3655	0.047*	
H9	0.2298	0.9753	0.3105	0.049*	
N1	0.7663 (2)	0.8046 (2)	0.40729 (19)	0.0497 (5)	
N2	0.7338 (2)	0.9065 (2)	0.37229 (17)	0.0424 (4)	
N3	0.2190 (2)	0.61730 (19)	0.43869 (17)	0.0405 (4)	
O1	0.5640 (2)	1.08767 (18)	0.30610 (17)	0.0553 (4)	
O2	0.3035 (2)	0.42371 (19)	0.29865 (18)	0.0590 (5)	
O3	0.0727 (2)	0.37090 (18)	0.41924 (17)	0.0571 (5)	
S1	0.15828 (7)	0.45175 (6)	0.34923 (5)	0.04300 (19)	

### Atomic displacement parameters (Å^2^)

**Table:**

	*U*^11^	*U*^22^	*U*^33^	*U*^12^	*U*^13^	*U*^23^
C1	0.0504 (13)	0.0580 (14)	0.0400 (12)	0.0181 (11)	0.0053 (10)	0.0081 (10)
C10	0.0355 (10)	0.0370 (10)	0.0364 (10)	0.0126 (8)	0.0059 (8)	0.0128 (8)
C11	0.0286 (9)	0.0394 (10)	0.0318 (9)	0.0119 (8)	0.0019 (7)	0.0076 (8)
C12	0.0331 (10)	0.0369 (10)	0.0316 (9)	0.0140 (8)	0.0005 (8)	0.0076 (8)
C13	0.0416 (12)	0.0485 (12)	0.0483 (12)	0.0224 (10)	0.0022 (9)	0.0177 (10)
C14	0.0285 (11)	0.0816 (18)	0.0628 (15)	0.0110 (11)	0.0078 (10)	0.0330 (14)
C15	0.0690 (17)	0.0516 (14)	0.0642 (16)	0.0189 (13)	0.0112 (13)	0.0293 (13)
C16	0.105 (3)	0.093 (3)	0.071 (2)	0.032 (2)	0.0053 (19)	0.0394 (19)
C17A	0.038 (2)	0.072 (4)	0.064 (4)	0.016 (3)	0.014 (2)	0.037 (4)
C17B	0.059 (5)	0.059 (5)	0.073 (6)	0.024 (5)	0.023 (4)	0.043 (5)
C18A	0.117 (6)	0.114 (6)	0.070 (4)	0.022 (4)	0.018 (3)	0.044 (4)
C18B	0.074 (6)	0.114 (9)	0.049 (5)	0.037 (6)	0.013 (5)	0.021 (6)
C19	0.099 (3)	0.098 (3)	0.0555 (17)	0.038 (2)	−0.0175 (17)	0.0068 (17)
C2	0.0527 (14)	0.0640 (16)	0.0520 (14)	0.0207 (12)	0.0015 (11)	0.0103 (12)
C3	0.0698 (17)	0.0534 (14)	0.0446 (13)	0.0157 (13)	−0.0038 (12)	0.0089 (11)
C4	0.088 (2)	0.0736 (19)	0.0408 (13)	0.0338 (17)	0.0084 (13)	0.0049 (13)
C5	0.0667 (17)	0.0604 (16)	0.0495 (14)	0.0285 (13)	0.0133 (12)	0.0093 (12)
C6	0.0446 (12)	0.0341 (10)	0.0426 (11)	0.0087 (9)	0.0063 (9)	0.0107 (9)
C7	0.0329 (10)	0.0345 (10)	0.0337 (10)	0.0099 (8)	0.0028 (8)	0.0090 (8)
C8	0.0285 (10)	0.0442 (11)	0.0453 (11)	0.0129 (8)	0.0052 (8)	0.0146 (9)
C9	0.0354 (11)	0.0448 (11)	0.0488 (12)	0.0200 (9)	0.0048 (9)	0.0190 (10)
N1	0.0374 (10)	0.0607 (12)	0.0561 (12)	0.0245 (9)	0.0037 (8)	0.0201 (10)
N2	0.0287 (9)	0.0521 (11)	0.0468 (10)	0.0156 (8)	0.0046 (7)	0.0160 (8)
N3	0.0408 (10)	0.0393 (10)	0.0401 (10)	0.0109 (8)	0.0068 (8)	0.0144 (8)
O1	0.0415 (9)	0.0510 (10)	0.0749 (12)	0.0130 (7)	0.0121 (8)	0.0259 (9)
O2	0.0538 (10)	0.0526 (10)	0.0732 (12)	0.0269 (8)	0.0122 (9)	0.0164 (9)
O3	0.0664 (11)	0.0446 (9)	0.0658 (11)	0.0143 (8)	0.0097 (9)	0.0303 (8)
S1	0.0451 (3)	0.0360 (3)	0.0494 (3)	0.0139 (2)	0.0067 (2)	0.0166 (2)

### Geometric parameters (Å, °)

**Table:**

C1—H1	0.9300	C18B—C17B	1.345 (12)
C1—C2	1.382 (4)	C19—H19C	0.9600
C10—O1	1.376 (3)	C19—H19B	0.9600
C10—C9	1.372 (3)	C19—H19A	0.9600
C11—C10	1.412 (3)	C2—H2	0.9300
C11—C12	1.401 (3)	C2—C3	1.388 (4)
C11—N2	1.366 (3)	C3—C19	1.506 (4)
C12—C13	1.417 (3)	C3—C4	1.374 (4)
C13—H13	0.9300	C4—H4	0.9300
C13—N1	1.317 (3)	C5—H5	0.9300
C14—H14B	0.9700	C5—C4	1.378 (4)
C14—H14A	0.9700	C6—C1	1.381 (3)
C14—C17A	1.546 (7)	C6—C5	1.380 (3)
C14—C17B	1.453 (8)	C7—N3	1.436 (3)
C15—H15B	0.9700	C7—C12	1.410 (3)
C15—H15A	0.9700	C7—C8	1.370 (3)
C15—C16	1.483 (4)	C8—H8	0.9300
C16—H16C	0.9600	C8—C9	1.408 (3)
C16—H16B	0.9600	C9—H9	0.9300
C16—H16A	0.9600	N2—C14	1.443 (3)
C17A—H17A	0.9300	N2—N1	1.358 (3)
C17A—C18A	1.335 (8)	N3—H3	0.84 (3)
C17B—H17B	0.9300	O1—C15	1.403 (3)
C18A—H18B	0.9300	S1—C6	1.767 (2)
C18A—H18A	0.9300	S1—N3	1.6280 (19)
C18B—H18D	0.9300	S1—O3	1.4369 (17)
C18B—H18C	0.9300	S1—O2	1.4260 (18)
			
C2—C1—H1	120.3	C17A—C18A—H18B	120.0
C6—C1—H1	120.3	C17A—C18A—H18A	120.0
C6—C1—C2	119.5 (2)	H18C—C18B—H18D	120.0
O1—C10—C11	117.06 (18)	C17B—C18B—H18D	120.0
C9—C10—C11	116.67 (19)	C17B—C18B—H18C	120.0
C9—C10—O1	126.26 (19)	C3—C2—H2	119.5
C12—C11—C10	122.13 (18)	C1—C2—H2	119.5
N2—C11—C10	131.2 (2)	C1—C2—C3	121.1 (3)
N2—C11—C12	106.64 (18)	C2—C3—C19	120.9 (3)
C7—C12—C13	136.1 (2)	C4—C3—C19	120.9 (3)
C11—C12—C13	104.30 (18)	C4—C3—C2	118.2 (3)
C11—C12—C7	119.59 (18)	C5—C4—H4	119.1
C12—C13—H13	124.3	C3—C4—H4	119.1
N1—C13—H13	124.3	C3—C4—C5	121.7 (3)
N1—C13—C12	111.4 (2)	C6—C5—H5	120.3
H14A—C14—H14B	108.8	C4—C5—H5	120.3
C17A—C14—H14B	110.7	C4—C5—C6	119.4 (3)
C17B—C14—H14B	86.7	C1—C6—S1	120.01 (17)
N2—C14—H14B	110.7	C5—C6—S1	119.80 (19)
C17A—C14—H14A	110.7	C5—C6—C1	120.2 (2)
C17B—C14—H14A	108.8	C12—C7—N3	120.09 (18)
N2—C14—H14A	110.7	C8—C7—N3	121.55 (18)
C17B—C14—C17A	27.3 (3)	C8—C7—C12	118.23 (19)
N2—C14—C17A	105.1 (3)	C9—C8—H8	119.3
N2—C14—C17B	127.8 (5)	C7—C8—H8	119.3
H15A—C15—H15B	107.9	C7—C8—C9	121.47 (19)
C16—C15—H15B	109.2	C8—C9—H9	119.1
O1—C15—H15B	109.2	C10—C9—H9	119.1
C16—C15—H15A	109.2	C10—C9—C8	121.89 (19)
O1—C15—H15A	109.2	C13—N1—N2	106.45 (17)
O1—C15—C16	112.1 (3)	C11—N2—C14	129.6 (2)
H16B—C16—H16C	109.5	N1—N2—C14	119.12 (19)
H16A—C16—H16C	109.5	N1—N2—C11	111.18 (18)
C15—C16—H16C	109.5	S1—N3—H3	110.4 (19)
H16A—C16—H16B	109.5	C7—N3—H3	116.7 (18)
C15—C16—H16B	109.5	C7—N3—S1	120.46 (15)
C15—C16—H16A	109.5	C10—O1—C15	118.87 (19)
C14—C17A—H17A	118.9	N3—S1—C6	107.93 (10)
C18A—C17A—H17A	118.9	O3—S1—C6	108.09 (11)
C18A—C17A—C14	122.3 (7)	O2—S1—C6	107.73 (11)
C14—C17B—H17B	122.8	O3—S1—N3	104.80 (10)
C18B—C17B—H17B	122.8	O2—S1—N3	108.66 (11)
C18B—C17B—C14	114.3 (9)	O2—S1—O3	119.18 (11)
H18A—C18A—H18B	120.0		

### Hydrogen-bond geometry (Å, °)

**Table:**

Cg1 is the centroid of the C7–C12 ring.

**Table:**

*D*—H···*A*	*D*—H	H···*A*	*D*···*A*	*D*—H···*A*
N3—H3···O3^i^	0.86 (2)	2.15 (2)	3.002 (2)	171 (2)
C5—H5···O2	0.93	2.53	2.908 (3)	104
C14—H14B···O1	0.97	2.35	2.974 (2)	121
C19—H19C···Cg1^ii^	0.96	2.87	3.622 (2)	136

Symmetry codes: (i) −*x*, −*y*+1, −*z*+1; (ii) −*x*, −*y*+1, −*z*.
